# Distal renal tubular acidosis in a patient with Hashimoto’s thyroiditis: a case report

**DOI:** 10.11613/BM.2023.020802

**Published:** 2023-06-15

**Authors:** Nontembiso Mhlana, Marizna Korf, Mogamat Razeen Davids, Mogamat-Yazied Chothia

**Affiliations:** 1 Division of Nephrology, Department of Medicine, Faculty of Medicine and Health Sciences, Stellenbosch University, Cape Town, South Africa; 2 Department of Chemical Pathology, Faculty of Medicine and Health Sciences, Stellenbosch University and National Health Laboratory Services, Cape Town, South Africa

**Keywords:** renal tubular acidosis, Hashimoto’s thyroiditis, hypokalaemia, rhabdomyolysis

## Abstract

Renal tubular acidosis (RTA) is a rare disorder that can be inherited or acquired, and results in an inability of the kidneys to maintain normal acid-base balance. We present a case of recurrent, severe hypokalaemia and rhabdomyolysis in a young woman who had an associated normal anion gap metabolic acidosis and was subsequently diagnosed with distal RTA associated with Hashimoto’s thyroiditis. Distal RTA associated with Hashimoto’s thyroiditis is rare and probably develops because of autoimmune-mediated mechanisms, causing an inability of the H^+^-ATPase pump in alpha-intercalated cells of the cortical collecting duct to secrete H^+^, with subsequent failure of urinary acidification. In this case, this hypothesis was supported by the exclusion of common genetic mutations associated with distal RTA. We illustrate that utilizing a systematic, physiology-based approach for challenging electrolyte and acid-base disorders enables identification of the root cause and underlying disease mechanisms.

## Introduction

Renal tubular acidosis (RTA) is a rare disorder that can be inherited or acquired, and results in an inability of the kidneys to maintain normal acid-base balance. The incidence and prevalence is largely unknown; however, a recent study reported a prevalence of 0.46 *per* 10,000 persons (*1*). Four types of RTA have been described, namely type 1 (distal), type 2 (proximal), type 3 (proximal and distal) and type 4 (hyperkalaemic type) (*2*). Conditions affecting the proximal tubule result in an inability to reabsorb filtered bicarbonate, while conditions affecting the connecting tubule and cortical collecting duct (CCD) result in an inability to excrete acid and hence generate new bicarbonate (*3*). This may result from an inability to secrete either ammonia (NH_3_) or H^+^, with subsequent failure to excrete sufficient acid in the form of ammonium (NH_4_^+^) (*3*).

We present a case of recurrent, severe hypokalaemia and rhabdomyolysis in a young woman who had an associated normal anion gap metabolic acidosis and was subsequently diagnosed with distal RTA associated with Hashimoto’s thyroiditis, and discuss the diagnostic and therapeutic challenges that were confronted.

The patient gave written, informed consent to publish and was approved by the Health Research Ethics Committee (HREC) of Stellenbosch University (HREC reference number: C22/10/033; Project identification number: 26729).

## Case presentation

A 19-year-old woman with no previous medical history presented to her local hospital with severe generalized weakness which started 3-4 days earlier. There was no previous history of similar episodes. The weakness was not preceded by strenuous exercise or a large carbohydrate meal. She denied drinking alcohol, smoking, or using recreational drugs. She also denied the use of diuretics or laxatives. Other than revealing proximal muscle weakness, the clinical examination was unremarkable. Blood pressure was 117/65 mmHg. There were no clinical features suggestive of systemic lupus erythematosus or Sjogren’s syndrome. Laboratory investigations revealed hypokalaemia, a normal anion gap metabolic acidosis and an elevated creatine kinase (Table 1). The urine biochemistry revealed an alkaline urine and a urine potassium-to-creatinine (K/Cr) ratio of 18.2, indicative of renal potassium wasting. The urine sodium concentration was not measured on this initial sample; therefore, the urine net charge could not be calculated. Glycosuria was absent. She was treated with intravenous potassium chloride and had a good response to therapy.

**Table 1:** Blood and urine biochemistry at first and second presentations

		**Initial presentation**		**Second presentation three months later**	
**Parameter, unit**	**Normal range**	**Blood**	**Urine**	**Blood**	**Urine**
Na, mmol/L	136-145	141	–	137	101
K, mmol/L	3.5-5.1	1.6	63.6	1.6	17.6
Urea, mmol/L	2.1-7.1	4.3	–	3.8	–
CREA, µmol/L	49-90	67	3500	64	1200
K/Cr ratio	< 1.5 during hypokalaemia	–	18.2	–	14.7
Cl, mmol/L	98-107	116	164	117	115
Osmolality, mOsm/kg	275-295 (serum) 50-1200 (urine)	296	435	297	269
CK, IU/L	20-180	15,390	–	1185	–
pH	7.35-7.45 (serum)	7.20	8.0	7.29	7.5
HCO_3_^−^, mmol/L	23-29	16	–	17	–
Alb, g/L	35-52	44	–	40	–
Alb-corrected AG*, mmol/L	9-16	9.0	–	4.0	–
Urine net charge, mmol/L	–	–	–	–	+ 3.6
TSH, pmol/L	0.51-4.30	–	–	26.5	–
fT4, pmol/L	12.6-21.0	–	–	9.4	–
*Alb-corrected anion gap (AG) = AG + 0.25 x (44 – measured albumin [in g/L]). Na – sodium. K – potassium. CREA – creatinine. K/Cr ratio – potassium to creatinine (K/Cr) ratio. Cl – chloride. CK – creatine kinase. HCO_3_^-^ – bicarbonate. Alb – albumin. TSH - thyroid-stimulating hormone. fT4 - free thyroxine.					

Three months later, the patient returned with the same clinical picture and laboratory profile (Table 1). In addition, the urine net charge (Na + K - Cl) was + 3.6 mmol/L, indicating the absence of urinary NH_4_^+^ excretion. A diagnosis of renal tubular acidosis (RTA) was made. Again, she had a good response to intravenous potassium supplementation. On further enquiry, she denied having a dry mouth, itchy eyes, arthralgia or dyspnoea, but reported unusual cravings for sour foods, particularly lemons. Physical examination revealed normal vital signs, she did not have a goitre, and cardiopulmonary evaluation was unremarkable. Her neurological evaluation revealed decreased muscle strength in upper and lower extremities, both proximally and distally. The tendon reflexes were decreased throughout; however, sensation was intact. Further investigation revealed elevated thyroid stimulating hormone and a low free thyroxine level, with positive anti-thyroglobulin and anti-thyroid peroxidase antibodies in keeping with Hashimoto’s thyroiditis. Other tests for autoimmune disorders were negative including anti-nuclear antibodies, anti-double stranded DNA antibodies, anti-SS-A (Ro) and anti-SS-B (La) antibodies. She was initiated on levothyroxine as well as potassium chloride supplementation and was subsequently referred to our centre for further diagnostic investigation.

## Further investigations

**Figure 1:**
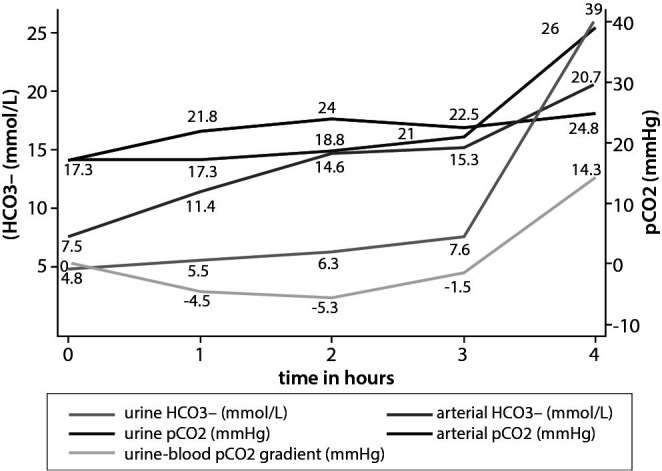
Hourly blood and urine sample results during the 4-hour sodium bicarbonate loading test. HCO_3_^-^ – bicarbonate. pCO_2_ - partial pressure of carbon dioxide.

We performed an intravenous sodium bicarbonate (NaHCO_3_) loading test for two reasons: (*1*) to distinguish between proximal and distal RTA, and (*2*) in the case of distal RTA, to identify whether there was failure of alpha-intercalated cells to pump H^+^ into the lumen of the distal nephron. Prior to performing the test, additional oral potassium chloride was administered to correct hypokalaemia. Following the administration of 10 mL of 10% calcium gluconate infusion, an infusion of 4.2% NaHCO_3_ was started at a rate of 160 mL/h (2.7 mL/kg/h) to alkalinize the urine to a target pH of 7.5. Hourly blood and urine samples were collected. At 4 hours, the urine pH target was reached (Figure 1). The calculated fractional excretion of bicarbonate was only 0.78%, eliminating the diagnosis of proximal RTA and therefore supporting a diagnosis of distal RTA. The calculated urine-blood PCO_2_ gradient was 14 mmHg (normal > 30 mmHg), suggesting that the mechanism of the distal RTA was failure of alpha-intercalated cells of the distal nephron to pump H^+^ into the lumen (Table 2).

**Table 2:** Blood and urine biochemistry at baseline and 4-hours following sodium bicarbonate loading test

		**Baseline**		**4 hours**	
**Parameter, unit**	**Normal range**	**Blood**	**Urine**	**Blood**	**Urine**
Na, mmol/L	136-145	135	155	138	117
K, mmol/L	3.5-5.1	5.9	105	3.5	100
Urea, mmol/L	2.1-7.1	4	107	4.2	237
CREA, µmol/L	49-90	66	4300	54	8700
Cl, mmol/L	98-107	112	274	103	179
Glc, mmol/L	5.6-6.9 (serum) 0 (urine)	–		6.2	0.4
Osmolality, mOsm/kg	275-295 (serum) 50-1200 (urine)	290	623	292	706
NH_4_^+^, mmol/L	–	–	0.7	–	0.8
Urine osmolal gap, mmol/L	–	–	- 4	–	35
Urine net charge, mmol/L	–	–	- 14	–	38
HCO_3_^–^, mmol/L	23-29	7.0	4.3	20.7	26.0
pH	7.35-7.45 (serum)	7.22	6.77	7.51	7.52
Urine pCO_2_, mmHg		–	17.3		39.0
Arterial pCO_2_, mmHg	35-45	17.3	–	24.7	
U-B pCO_2_ gradient, mmHg	> 30	0		14.3	
Fractional excretion of HCO_3_^–^, %	< 5 > 15 (Proximal RTA)	–	0.94	–	0.78
U-B pCO_2_ - urine-blood partial pressure of carbon dioxide. RTA - renal tubular acidosis. Na – sodium. K – potassium. CREA – creatinine. Cl – chloride. Glc – glucose. HCO_3_^-^ – bicarbonate. NH_4_^+^ - ammonium.					

We also performed genetic testing for inherited mutations causing distal RTA because the patient mentioned that her father had similar complaints; however, no potentially pathogenic variants were identified.

Our patient was informed that no genetic mutations were identified. She had a good response to oral potassium chloride, oral sodium bicarbonate and levothyroxine replacement. She was also advised to continue consuming lemons.

## Methods

Blood samples for laboratory testing were obtained using a serum separation tube (BD Vacutainer, Becton Dickinson, Wokingham, UK), while blood gas samples were collected via a butterfly needle into a pre-filled, spray-dried calcium-balanced heparin syringe, specifically the BD A-line syringe (also manufactured by BD Vacutainer, Becton Dickinson, Wokingham, UK). The Roche Cobas 6000 analyser (Roche Diagnostics, Mannheim, Germany) was used to measure concentrations of Na, K, Cl, urea and CREA. OsmoTECH Single-Sample Micro-Osmometer (Advanced Instruments, Massachusetts, USA) was used to measure urine osmolality. The plasma ammonia assay on the Roche Cobas 6000 analyser was used to determine urine NH_4_^+^ concentration. Arterial blood and urine samples were collected at baseline to measure pH (arterial), HCO_3_^–^ and partial pressure of carbon dioxide (pCO_2_) using the GEM Premier 5000 from Instrumentation Laboratory Company (Massachusetts, USA). Urine pH was measured at the bedside using a Beckman Coulter pHi 520 pH-meter. Serum creatine kinase, albumin, thyroid stimulating hormone, free thyroxine, anti-thyroglobulin, and anti-thyroid peroxidase were measured on the Roche Cobas 6000 analyser (Roche Diagnostics, Mannheim, Germany). Manual enzyme immunoassays from Bio-Rad Laboratories Inc. (California, USA) were used to perform tests for anti-double stranded DNA, anti-SS-A, and anti-SS-B antibodies. A manual indirect fluorescent antibody assay from Bio-Rad Laboratories Inc. (California, USA) was used to test for anti-nuclear antibodies.

The genetic testing was carried out using the Invitae diagnostic testing. A saliva sample was obtained and analysed using the Invitae renal tubular disorders panel, which examines 39 genes, including ATPV0A4, ATP6V1B1, FOXI1, SLC4A1, and SLC4A4, for sequence analysis and deletion/duplication testing.

## Discussion

We present a case of recurrent, severe hypokalaemia and rhabdomyolysis in a young woman who had an associated normal anion gap metabolic acidosis. The first step was to determine if the patient was in any imminent danger related to hypokalaemia. Due to the muscle weakness, we were concerned about respiratory muscle involvement; therefore prompt treatment with intravenous K^+^ was initiated to avoid this. As our patient had recurrent hypokalaemia, intracellular shift of K^+^ due to periodic paralysis was initially considered; however, we thought this unlikely because of the associated acid-base disturbance and the obvious renal K^+^ wasting indicated by the high urinary K/Cr ratios. This also made gastrointestinal losses of K^+^ unlikely.

Renal K^+^ wasting due to hyperaldosteronism or diuretic abuse were unlikely since our patient was normotensive and had metabolic acidosis rather than metabolic alkalosis. We believe that the excessive K^+^ excretion in the CCD was due to the more negatively charged lumen, related to the bicarbonaturia.

To establish a diagnosis of RTA, urine NH_4_^+^ excretion should be estimated. The normal renal response to an acidosis is to increase the excretion of NH_4_^+^, which can be as much as 200 mmol *per* day (*3*). There remains controversy on the best method for estimating urine NH_4_^+^ excretion. We prefer using the urine osmolal gap because the urine net charge assumes, sometimes erroneously, that NH_4_^+^ is only excreted with chloride (*3*). A previous study reported a strong correlation between the urine osmolal gap and measured urine NH_4_^+^ concentration, while the urine net charge performed poorly (*4*). Urine NH_4_^+^ can also be measured directly on auto-analysers using the plasma ammonia assay after predilution of the urine specimen (*5*). In our patient, the urine osmolal gap indicated absent urine NH_4_^+^ excretion, supporting a diagnosis of RTA.

Diseases affecting the proximal tubule will result in an inability to reabsorb filtered bicarbonate (proximal or type 2 RTA), while diseases affecting the distal nephron may result in failure to excrete acid in the form of NH_4_^+^ salts (distal or type 1 RTA). Following NaHCO_3_ loading, the fractional excretion of bicarbonate was only 0.78%, eliminating proximal RTA (*6*). Therefore, we concluded that distal RTA was present.

The NaHCO_3_ loading test was also used to probe the mechanism causing the distal RTA. At a urine pH of 7.5, the urine-blood PCO_2_ difference was 14 mmHg, indicating a failure of the H^+^-ATPase pump to secrete sufficient H^+^. A previous study reported that at a urine-blood PCO_2_ difference cut-off value of 30 mmHg, the sensitivity and specificity was 100% for a defect involving the H^+^-ATPase pump (*7*). The expression of other targets in alpha-intercalated cells has also been shown to be affected in distal RTA and include the anion exchanger-1 located in the apical membrane, pendrin in the basolateral membrane and intracellular carbonic anhydrase-II (*8*). Failure of these channels inhibit H^+^-ATPase action by causing intracellular alkalosis.

Few case reports have reported on the exclusive association between distal RTA and Hashimoto’s thyroiditis (*9*-*13*). Females were mostly affected, and at an average age of 30 years. It is speculated that autoantibodies directed toward the H^+^-ATPase pump, among others, in the apical membranes of alpha-intercalated cells may be responsible (*8*). Most studies regarding autoantibody mechanisms of distal RTA have been conducted in patients with Sjogren’s syndrome (*7*, *8*). Studies that performed immunohistochemical staining have reported absent H^+^-ATPase pump expression (*7*, *14*, *15*). Since we did not identify any known genetic mutations resulting in inherited forms of distal RTA, an autoimmune-mediated mechanism was postulated.

Treatment mainly involves correcting hypokalaemia and metabolic acidosis, as well as thyroxine replacement. A recent case report reported dramatic improvement of hypokalaemia and metabolic acidosis within two weeks of potassium citrate and oral prednisone therapy, adding further support to an autoimmune mechanism (*16*). Our patient responded well to oral potassium chloride, oral sodium bicarbonate and levothyroxine replacement. In addition, our patient had a peculiar craving for lemons, particularly when she experienced leg cramps, which subsided following consumption. Since lemons contain potassium citrate, this may also be considered adjunctive therapy.

One of the study’s limitations was the use of the plasma ammonia assay on the Roche Cobas 6000 analyser to measure urine NH_4_^+^ concentrations, which is not an officially validated method for urine analysis in the laboratory (*5*). The study could have benefited from additional testing, such as immunohistochemical staining of kidney tissue for the H^+^-ATPase pump, as demonstrated by other studies (*14*, *15*).

In conclusion, distal RTA, which is linked to Hashimoto’s thyroiditis, is rare. The prevailing theory suggests that an autoimmune-mediated mechanism may be the cause, resulting in the inability of the H^+^-ATPase pump in alpha-intercalated cells of the CCD to secrete H^+^ and subsequent failure in urinary acidification. In this case, this hypothesis has been supported by the exclusion of common genetic mutations associated with distal RTA.

## Data Availability

All data generated and analysed in the presented study are included in this published article (and its supplementary files).

## References

[1] Bianic F Guelfucci F Robin L Martre C Game D Bockenhauer D. Epidemiology of Distal Renal Tubular Acidosis: A Study Using Linked UK Primary Care and Hospital Data. Nephron. 2021;145:486–95. 10.1159/000516876 34198293

[2] Palmer BF Kelepouris E Clegg DJ. Renal tubular acidosis and management strategies: a narrative review. Adv Ther. 2021;38:949–68. 10.1007/s12325-020-01587-5 33367987 PMC7889554

[3] Kamel KS, Halperin ML, editors. Fluid, Electrolyte and Acid-Base Physiology - A Problem-Based Approach. 5th ed. Philadelphia: Elsevier Saunders; 2016. 10.1016/B978-0-323-35515-5.00001-4 10.1016/B978-0-323-35515-5.00001-4

[4] Goldstein MB Bear R Richardson R Marsden P Halperin M. The urine anion gap: a clinically useful index of ammonium excretion. Am J Med Sci. 1986;292:198–202. 10.1097/00000441-198610000-00003 3752165

[5] Ha LY Chiu WW Davidson JS. Direct urine ammonium measurement: time to discard urine anion and osmolar gaps. Ann Clin Biochem. 2012;49:606–8. 10.1258/acb.2012.012013 23038701

[6] Han JS Kim G-H Kim J Jeon US Joo KW Na KY Secretory-defect distal renal tubular acidosis is associated with transporter defect in H+-ATPase and anion exchanger-1. J Am Soc Nephrol. 2002;13:1425–32. 10.1097/01.ASN.0000013882.73122.2B 12039970

[7] Kim S Lee JW Park J Na KY Joo KW Ahn C The urine-blood PCO2 gradient as a diagnostic index of H+-ATPase defect distal renal tubular acidosis. Kidney Int. 2004;66:761–7. 10.1111/j.1523-1755.2004.00801.x 15253731

[8] Ungureanu O Ismail G. Distal Renal Tubular Acidosis in Patients with Autoimmune Diseases—An Update on Pathogenesis, Clinical Presentation and Therapeutic Strategies. Biomedicines. 2022;10:2131. 10.3390/biomedicines10092131 36140232 PMC9496140

[9] Finn BC Young P Bruetman JE Forrester M Lombi F Campolo Girard V. Hypokalemia, distal renal tubular acidosis, and Hashimoto’s thyroiditis. Nefrología. 2008;28:569–70. 18816228

[10] Naveen L Malkarnekar S. Adult-onset distal renal tubular acidosis with hypokalemic quadriparesis in a patient with autoimmune hypothyroidism. J Integr Nephrol Androl. 2014;1:82–4. 10.4103/2225-1243.143392

[11] Koul PA Wahid A. Distal renal tubular acidosis and hypokalemic paralysis in a patient with hypothyroidism. Saudi J Kidney Dis Transpl. 2011;22:1014–6. 21912036

[12] Meregildo-Rodríguez ED Failoc-Rojas VE. Case report: recurrent hypokalemic periodic paralysis associated with distal renal tubular acidosis (type 1) and hypothyroidism secondary to Hashimoto’s thyroiditis. F1000Res. 2018;7:1154. 10.12688/f1000research.15662.1 30647907 PMC6325611

[13] Mason AM Golding P. Renal tubular acidosis and autoimmune thyroid disease. Lancet. 1970;2:1104–7. 10.1016/S0140-6736(70)92296-8 4097906

[14] Cohen EP Bastani B Cohen MR Kolner S Hemken P Gluck SL. Absence of H (+)-ATPase in cortical collecting tubules of a patient with Sjogren’s syndrome and distal renal tubular acidosis. J Am Soc Nephrol. 1992;3:264–71. 10.1681/ASN.V32264 1391725

[15] Walsh S Turner CM Toye A Wagner C Jaeger P Laing C Immunohistochemical comparison of a case of inherited distal renal tubular acidosis (with a unique AE1 mutation) with an acquired case secondary to autoimmune disease. Nephrol Dial Transplant. 2007;22:807–12. 10.1093/ndt/gfl662 17205967

[16] Wang S Dong B Wang C Lu J Shao L. From Bartter’s syndrome to renal tubular acidosis in a patient with Hashimoto’s thyroiditis: A case report. Clin Nephrol. 2020;94:150. 10.5414/CN109820 32691728

